# Issues for patchy tissues: defining roles for gut-associated lymphoid tissue in neurodevelopment and disease

**DOI:** 10.1007/s00702-022-02561-x

**Published:** 2022-10-30

**Authors:** T. Abo-Shaban, S. S. Sharna, S. Hosie, C. Y. Q. Lee, G. K. Balasuriya, S. J. McKeown, A. E. Franks, E. L. Hill-Yardin

**Affiliations:** 1 School of Health and Biomedical Sciences, RMIT University, Bundoora, VIC Australia; 2 Department of Pathology and Immunology, Baylor College of Medicine, Houston, TX USA; 3 Department of Pathology, Texas Children’s Microbiome Center, Texas Children’s Hospital, Houston, TX USA; 4 Department of Physiology and Cell Biology, Kobe University School of Medicine, 7-5-1 Kusunoki-Cho, Chuo, Kobe, 650-0017 Japan; 5 Development and Stem Cells Program, Monash Biomedicine Discovery Institute, Department of Anatomy and Developmental Biology, Monash University, Clayton, VIC Australia; 6 Department of Physiology, Anatomy and Microbiology, School of Life Sciences, La Trobe University, Bundoora, VIC Australia

**Keywords:** Caecal patch, Peyer’s patch, Mice, Microbes, Neuroimmune response, Autism spectrum disorder, Autism

## Abstract

Individuals diagnosed with neurodevelopmental conditions such as autism spectrum disorder (ASD; autism) often experience tissue inflammation as well as gastrointestinal dysfunction, yet their underlying causes remain poorly characterised. Notably, the largest components of the body’s immune system, including gut-associated lymphoid tissue (GALT), lie within the gastrointestinal tract. A major constituent of GALT in humans comprises secretory lymphoid aggregates known as Peyer’s patches that sense and combat constant exposure to pathogens and infectious agents. Essential to the functions of Peyer’s patches is its communication with the enteric nervous system (ENS), an intrinsic neural network that regulates gastrointestinal function. Crosstalk between these tissues contribute to the microbiota-gut-brain axis that altogether influences mood and behaviour. Increasing evidence further points to a critical role for this signalling axis in neurodevelopmental homeostasis and disease. Notably, while the neuroimmunomodulatory functions for Peyer’s patches are increasingly better understood, functions for tissues of analogous function, such as caecal patches, remain less well characterised. Here, we compare the structure, function and development of Peyer’s patches, as well as caecal and appendix patches in humans and model organisms including mice to highlight the roles for these essential tissues in health and disease. We propose that perturbations to GALT function may underlie inflammatory disorders and gastrointestinal dysfunction in neurodevelopmental conditions such as autism.

## Introduction

The gut-brain axis is the well-established, bi-directional communication between the gut and brain. Classically, the gut-brain axis influences a variety of communication pathways such as the neuroendocrine system, immune system and the peripheral nervous system (parasympathetic, sympathetic and enteric nervous systems (ENS)). However, recent evidence has shifted the classical concept of the gut-brain axis to include the microbiota, with many studies highlighting the microbiota playing a mediatory role in these pathways. The microbiota-gut-brain axis modulates the body’s response to changes in the microbiota within the gastrointestinal (GI) tract, influencing gut function, bodily immune response, physiological stress and cognitive behaviour. Several studies indicate these complex interacting pathways are disturbed in neurodevelopmental disorders including autism spectrum disorder (ASD; autism) (Campbell et al. 2022; Matta et al. 2019; Mayer et al. 2014; Sharon et al. 2019; Morais et al. 2021; Hughes et al. 2018). Central to our understanding of the cellular basis for the microbiota-gut-brain axis is the gut-associated lymphoid tissue (GALT), a major component of the immune system, comprising multiple lymphoid masses located in the small intestine (i.e., Peyer’s patches), the appendix/caecum (i.e., caecal patch tissue) as well as in the large intestine and rectum. Given that the GI tract hosts countless microorganisms required for normal GI function, as well as pathogens and infectious agents, it is unsurprising that up to 70% of the body’s immune system is located within the gut (Jung et al. 2010). To maintain gut health, a dynamic regulatory system involving GALT-mediated activity is established during development which modulates the composition of gut microbes, amongst other functions.

Gut dysfunction is common in many neurodevelopmental disorders including autism; however, the aetiology is poorly understood. Examining effects of altered GALT structure and function in preclinical models of autism may lead to an increased understanding and define targets for drug intervention to improve GI symptoms. Due to limited access to biopsy tissue for example, it is difficult to fully characterise the impact of GALT dysfunction in humans. Therefore, we can investigate other mammalian species including transgenic rodent models to clarify whether the gut immune system is affected in neurodevelopmental disorders. Of particular interest is GALT found in the caecum in mice (the equivalent of the appendix in humans), which is altered in a mouse model of autism (Sharna et al. 2020). Here we compare the structure and function of caecum GALT and Peyer’s patches located throughout the small intestine of the mouse and human gut. We describe similarities and differences in these GALTs located in different regions of the GI tract to identify potential impacts on these tissues in neurodevelopmental disorders such as autism.

There is comparatively little known about caecal patch structure and function, yet, within these tissues lie specialized follicle-associated epithelial cells that overlay lymphoid tissue domes containing intestinal micro-fold or membranous epithelial cells, referred to as M cells. These M cells play a crucial role in the mucosal immune system through their roles in antigen sampling and transporting luminal antigens across the apical surface via their cytoplasm to underlying cells, leading to an immune response (Kraehenbuhl and Neutra 1992; Owen and Ermak 1990; Trier 1991).

When comparing the structure and function of Peyer’s and caecal patches in humans and mice, respectively, differences both macroscopic (i.e. size and characteristics of the surrounding tissue such as microvilli) and microscopic (such as the cellular composition and morphology of patches) are evident. Microbial populations are specific to gut regions and their interactions with surrounding environments likely influence the development of lymphoid aggregates in the small intestine and caecum of the host. As such, alterations in host, including changes in nervous system function and patterns of innervation, can influence the development and function of Peyer’s patches and caecal patch tissue. Furthermore, Liu and colleagues reported that host genetics could modify the microbiome (Liu et al. 2016; Goodrich et al. 2014), while recent compelling data indicate that modifications to gut microbial metabolites can lead to changes in brain structure and behaviour in the context of autism (Needham et al. 2022; Campbell et al. 2022). These recent studies altogether suggest that the microbiota-gut-brain axis, which includes the sensing of microbes and pathogens via GALT signalling, likely play important roles in neurodevelopmental disorders. To clarify the biological mechanisms underlying these complex signalling interactions, in-depth investigations of the bi-directional communication pathways between GALT and the nervous system are needed, in preclinical mouse models and beyond.

## Peyer’s and caecal patch structure and function

Peyer’s patches were first identified by the Swiss clinician and anatomist Johann Peyer. Peyer’s patches are randomly distributed along the length of the small intestine and are most densely populated in the ileum and lower jejunum in humans. They are typically oval shaped, measuring 3–4 cm in length. However, the size, shape and distribution of Peyer’s patches is highly variable, ranging from 0.03 to 17 cm^3^ in volume (Van Kruiningen et al. 2002). The number of Peyer’s patches peaks in adolescence and as many as 240 Peyer’s patches can be documented in the human small intestine, however, numbers decline with age (Cornes 1965a). Peyer’s patches are predominantly located on the anti-mesenteric border except in the distal 10–15 cm of the ileum where they are so densely distributed that they form a “ring-like” structure of lymphoid tissue close to the ileocecal valve (Van Kruiningen et al. 2002).

Peyer’s patches are critical for providing protection and maintaining tissue homeostasis within the gastrointestinal tract. In addition, these lymphoid aggregates act as entry points for pathogens, enabling the host to recognize them and to mount a subsequent immune response. One point of contention has been the presence of mucus overlying Peyer’s patch aggregates, thought to inhibit movement of pathogens toward the lymphoid tissue. But studies confirm its presence as a penetrable mucus layer that does not significantly hinder pathogens, contrary to what was first proposed (Ermund et al. 2013a, b; Ermund et al. 2013a, b). The human appendix is an inferior finger-like, blind-ended extension (approximately 5–10 cm long and 0.5–1 cm wide) of the caecum, at the junction between the small and large intestines. Contained within the appendix are multiple aggregates of lymphoid follicles located within the submucosa and lamina propria of the appendiceal wall (Spencer et al. 1985; Masahata et al. 2014; Kooij et al. 2016).

**Fig. 1:**
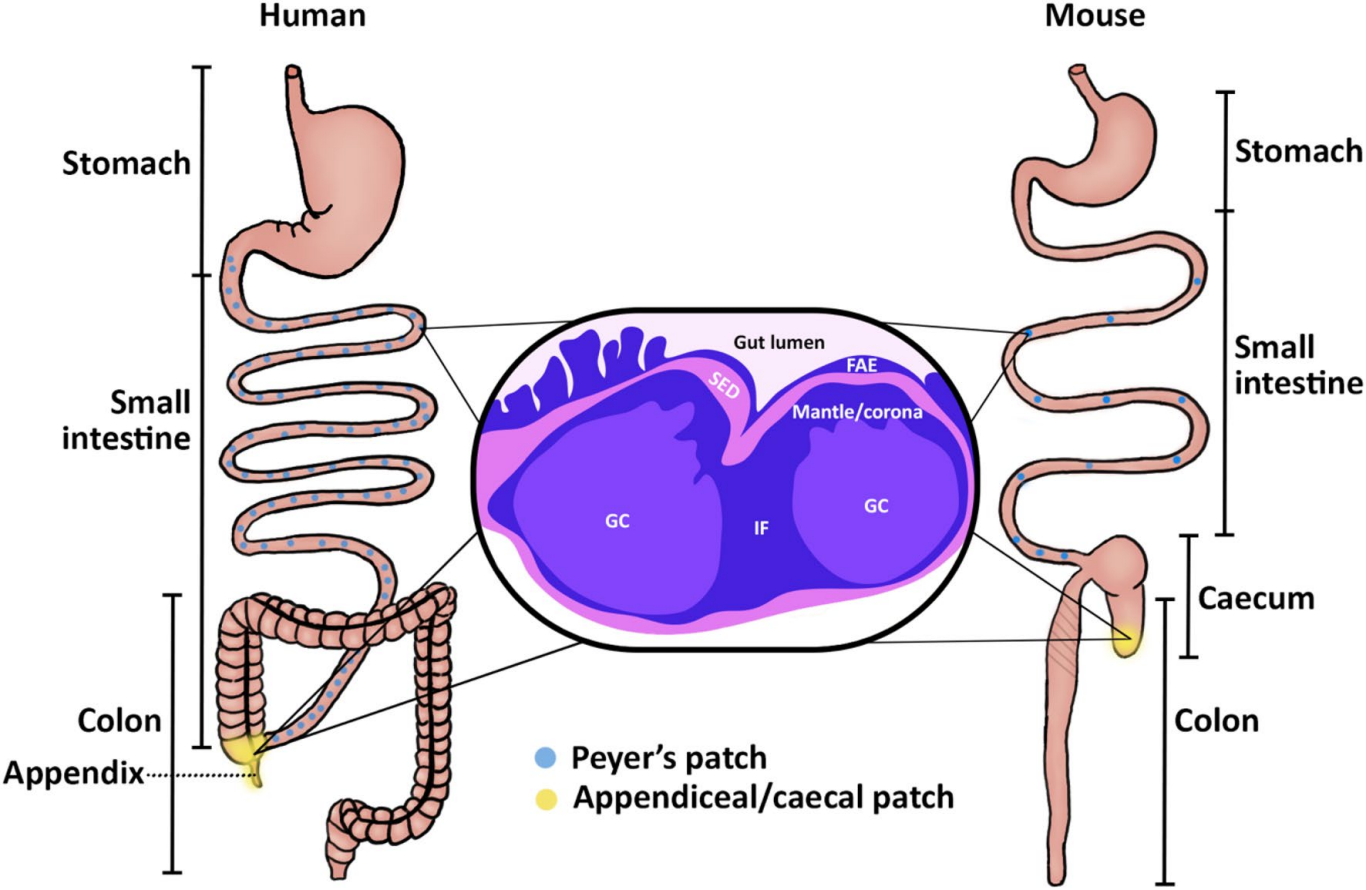
*Gut-associated lymphoid tissue (GALT) in human and mouse*. GALT comprises multiple lymphoid masses located in the small intestine (Peyer’s patches) and the appendix/caecum (appendiceal/caecal patches). In humans, Peyer’s patches are randomly distributed along the length of the small intestine, and aggregates of lymphoid follicles are located within the submucosa and lamina propria of the appendiceal wall. Mice have fewer Peyer’s patches compared to humans and are similarly distributed along the anti-mesenteric border of the small intestine. In mice, the caecum is the equivalent of the human appendix and typically contains a single large aggregate of lymphoid follicles near the tip of the caecum referred to as the ‘caecal patch’. Lymphoid tissue in the small intestine and appendix/caecum of both humans and mice are similar in microscopic structure. The follicles are positioned in close proximity to the luminal content in line with their role of maintaining intestinal immune homeostasis. Overlying the luminal surface of the follicles is specialized FAE, below which the SED lies, followed by an outer mantle/coronal layer of B cells, and the middle GC. Between follicles are IF regions which are rich in T cells. *FAE* follicle-associated epithelium, *SED* subepithelial dome, *GC* germinal centre, *IF* interfollicular region

In terms of macroscopic anatomy, mice have fewer and more uniformly sized Peyer’s patches than humans (Haley 2003). Like humans, however, Peyer’s patches are distributed along the anti-mesenteric border of the small intestine in mice. Mice typically have approximately 10 Peyer’s patches (De Jesus et al. 2013). The number of Peyer’s patches in mice is presently understood to be set at birth and remain constant (Cornes 1965a). In contrast with humans, Peyer’s patches in mice are distributed along the small intestine rather than the dense clusters at the ileo-caecal valve found in humans. In mice, the caecum is the equivalent of the human appendix and typically contains a single large aggregate of lymphoid follicles near the tip of the caecum within the caecal apex, referred to as the ‘caecal patch’ (Fig. 1).

### Peyer’s patch and caecal-appendix function

Peyer’s patches are targeted regions along the gastrointestinal tract that monitor and prevent the growth of pathogenic microbes in the gastrointestinal tract. The role of Peyer’s patches in microbial sensing is particularly highlighted as it can be a site for the accumulation of harmful proteinaceous infectious particles (prions) (Prinz et al. 2003). The very nature of being able to sense the environment of the gut leaves Peyer’s patches vulnerable to entry of invading prion proteins, which once they have penetrated the gut efficiently spread disease to the brain. The precise mechanism of how prions enter the Peyer’s patch is unknown and is another reason why this area of research is important.

The appendix was initially thought to be an evolutionary remnant from *Homo sapiens’* ancestors owing to their high-fibre diets. This suggestion stemmed from the plausible explanation that the equivalent intestinal compartment, the caecum, is proportionally enlarged in herbivores such as rabbits and cows (Furness et al. 2015; Smith et al. 2009; Nguyen et al. 2015). In many mammals, the caecum is a primary site for the production of short chain fatty acids (SCFAs) (den Besten et al. 2013; Zaborin et al. 2020), important by-products of bacterial fermentation of dietary foods (Nicholson et al. 2012; Roy et al. 2006). Currently, the mechanisms for neurally regulated gastrointestinal motility in the caecum remain poorly characterised. Nevertheless, a churning motility pattern in rabbit caecum is hypothesised to mix digesta with microbial content in addition to contributing to the process of fermentative digestion (Hulls et al. 2012, 2016). How the location and presence of caecal patches affect caecal motility, however, remains unclear.

The caecum has been suggested to act as a repository to provide a ‘reserve’ of bacteria in order to replenish commensal microbiota within the gastrointestinal tract in the event of inflammation, resulting in diarrhoea (Bollinger et al. 2007; Laurin et al. 2011). In terms of health outcomes in animals, a study of 258 species correlated extended life span with the presence of a caecal appendix (Collard et al. 2021). Relevant to this correlative finding, the caecal appendix has also been suggested to serve a role in anti-tumour formation and prevent death from fatal diarrhoea. On this, there is evidence suggesting that appendectomy correlates with increased colorectal cancer in patients who have chronic ulcerative colitis (Harnoy et al. 2016; Stellingwerf et al. 2019). In modern times, the requirement for caecal appendix functions that protect from fatal diarrhoea has been alleviated by improved antibiotics to treat this condition (Collard et al. 2021). The caecum may also play an essential role in optimizing exposure of the host to antigens via regional lymphoid tissue. There, a case of appendicitis could boost immune function and subsequent appendectomy may therefore have minimal influence on health (Collard et al. 2021).

Growing evidence suggests that specialised cell types within the caecal patch may influence the composition of commensal bacterial populations (Laurin et al. 2011). In mice, conserved microbial populations are largely embedded within the nutrient-rich mucus coating or biofilm that continually replenishes the gut microbiota. Different regions of the caecum support contrasting microbial populations, with *Proteobacteria* and *Deferribacteres* found at highest abundance in the caecal crypts and *Firmicutes* present at highest levels in the caecal lumen (Zaborin et al. 2020). As such, changes in the nervous system associated with neurodevelopmental disorders such as autism could modify neuroimmune interactions and microbial sensing within GALT as well as impacting microbial populations in local niches of the caecum within the GI tract.

### Cellular composition of Peyer’s and caecal patches

**Fig. 2:**
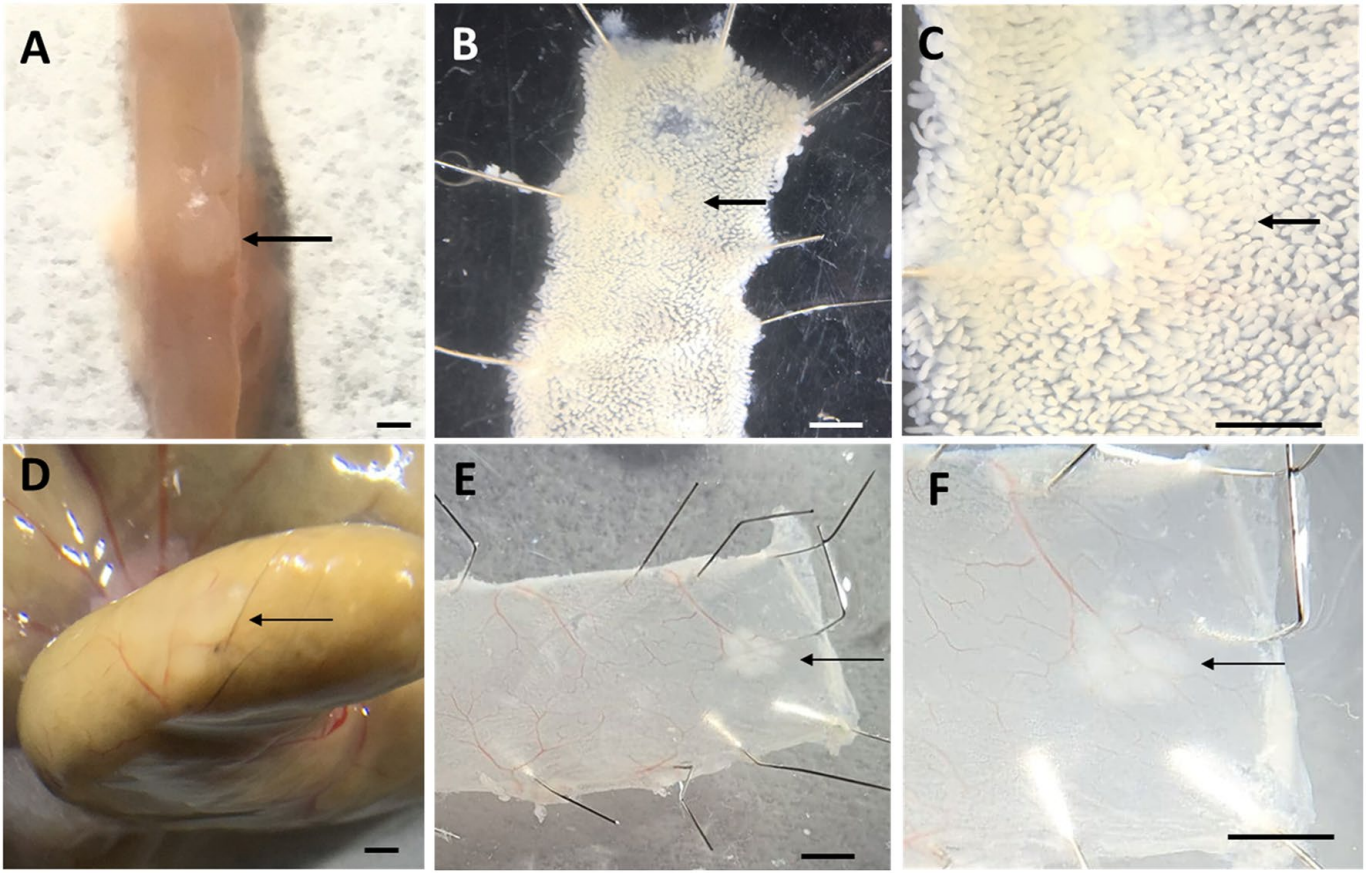
*Identification of Peyer’s patches and Caecal patch in adult mice* **A**–**C**, Peyer’s patch A, Peyer’s patch identifiable as a raised protrusion. **B**, **C** Multiple clusters of follicles can be clearly identified amongst the villi within the small intestine. **D** Caecal patch is not obvious externally. **E**, **F** Once opened, a caecal patch (identified by arrow) is clearly identified as a cluster of follicles at the terminal end of caecum. Scale bar: 0.2 mm

Both Peyer’s patch and caecal lymphoid aggregates are located within the submucosa and lamina propria of the gastrointestinal tract. The high cell density in these lymphoid aggregates, particularly in Peyer’s patches, forms protruding nodules which are visible from the peritoneal aspect of the dissected GI tract (Fig. 2). Histological staining of Peyer’s and caecal patch tissue sections (i.e., haematoxylin and eosin (H&E)) reveals subregions with different cellular composition and densities within these lymphoid tissue types (Cesta 2006; Jung et al. 2010; Spencer et al. 1985; Da Silva et al. 2017).

**Fig. 3:**
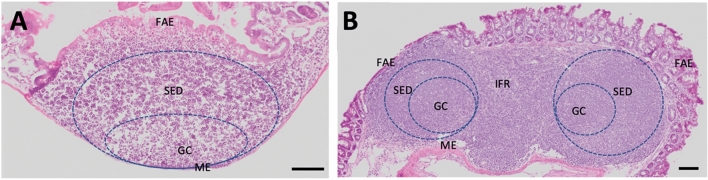
*Location of active zones within a Peyer’s and caecal patch in adult mice.* H&E stained transverse section of mouse **A** Peyer’s patch and **B** caecal patch. *GC* Germinal centre, *SED* Sub Epithelial Dome, *FAE* Follicular-associated Epithelium, *ME* Muscularis externae. Scale bar represents 100 µm

Both Peyer’s and caecal patch aggregates comprise a follicular area which contains between 6 and 12 lymphoid follicles in the Peyer’s patch (Haley 2003) and 8–14 in the caecal patch (Clark et al. 1994). Each lymphoid follicle has a germinal centre (GC) and a subepithelial dome (Gebert et al. 1992) that is covered by the follicle-associated epithelium (Fig. 3). The follicles are separated by less dense interfollicular regions (Cesta 2006). The germinal centre is densely packed with proliferating B lymphocytes, follicular dendritic cells and macrophages. The subepithelial dome comprises a mixed-cell zone containing B and T lymphocytes, macrophages and dendritic cells. The structural and functional properties of the follicular-associated epithelium differ to the mucosal epithelium of the GI tract. Notably, the follicular-associated epithelium is more porous, produces less mucus and contains specialised Microfold (M) cells which transcytose macromolecules such as soluble proteins, antigens, a process which connects bacteria and viruses from the intestinal lumen to immune cells within the host (Jung et al. 2010).

Previous comparisons between the Peyer’s patch and caecal patch in mice have focused primarily on the follicular-associated epithelium due to the presence of M cells in this region (Clark et al. 1994). In comparison to Peyer’s patch tissue, caecal patch follicle-associated epithelium has a higher concentration of mucus-secreting goblet cells (Bhalla and Owen 1982). In addition, M cells within the caecal patch have different morphological and biochemical characteristics to those seen in Peyer’s patches. Specifically, caecal patch M cells (in rabbits) have longer and more irregular microvilli than the surrounding enterocytes (Jepson et al. 1993) and lectin staining showed they express a different profile of specific glycans (Clark et al. 1994). The dome area of the caecal patch contains more immature M cells (which have lower ability to take up antigens (Kobayashi et al. 2019)) than the equivalent region of Peyer’s patches. Based on these findings, the dense population of caecal patch goblet cells may produce larger volumes of protective mucus at the caecal patch compared to the Peyer’s patch environment. These cellular differences could be driven by the greater number of microorganisms present in the caecal lumen compared to those encountered by Peyer’s patches in the lumen of the small intestine. Interestingly, it has been proposed that M cell density within the follicular-associated epithelium is dependent on the presence of bacteria (Jung et al. 2010).

## Development of Peyer’s patches and Caecal patches

### Human Peyer’s patch development

The development of Peyer’s patches commences at 11 weeks’ gestation, marked by the presence of CD45 + CD4 + CD3-HLA-DR + cells within the gastrointestinal tissue. These molecular markers, which identify a variety of progenitor immune cells, infiltrate the lamina propria of the small intestine (Spencer et al. 1986, 1985) and are understood to constitute cells functionally analogous to lymphoid tissue inducer (LTi) cells. Both human and mouse LTi cells induce adhesion molecules and recruit them to Peyer’s patches via IL-7Rɑ/IL2RG (γc)/JAK-3 pathways, an essential mechanism for patch development (Cupedo et al. 2009; Berteloot et al. 2021). At 14 weeks gestation, organised clusters of B- and T-lymphocytes become identifiable within the human small intestine and, by 19 weeks, such clusters develop into distinct but functionally immature Peyer’s patches that lack germinal centres (Cornes 1965b; Spencer et al. 1986). Functional maturation of Peyer’s patches subsequently occurs postnatally following colonisation of commensal bacteria (Cornes 1965b). It is noteworthy to mention that the majority of studies elucidating the cellular mechanisms for immune cell migration to the primordial Peyer’s patch during its formation have been conducted in mice models. Studies to substantiate or refute such processes occurring during the development of human Peyer’s patches remain to be undertaken. Indeed, improved knowledge of the development, structure and function of Peyer’s patches may lead to an improved understanding of the roles for these tissues in sustaining optimal lifelong gut health as well as possible roles in neurodevelopmental disorders such as autism.

### Human caecal appendix development

Within the caecal appendix, lymphocyte aggregates are first observed as early as week 17 of the developing human foetus and grow in number and size as the foetus reaches full term (Malas et al. 2004; Jones et al. 1972). In 1972, Jones and colleagues documented a pronounced increase in the number and size of lymphoid aggregates in neonates that die from infection compared to other causes (Jones et al. 1972), suggesting a role for this tissue in the immune response. Postnatally, appendiceal GALT persists throughout the human lifespan. The germinal centres of the aggregates are evident at approximately 1 month of age and rapidly develop within the first year of life. Atrophy of these germinal centres subsequently follows in adolescent years, during which senescence renders lymphoid follicles visibly smaller and fewer in number (Dasso et al. 2000; Gebbers and Laissue 2004). In addition, the development of human appendiceal lymphoid tissue also parallels bacterial translocation (that is, the movement of gut commensal bacteria to mesenteric lymph nodes) that occurs in the appendix (Gebbers and Laissue 2004). These observations, therefore, illustrate a temporal, developmental pattern for GALT in the appendix, how it is important in the first postnatal year, and how this tissue changes in adolescence. Given that neuro-immune interactions occur between the brain and the enteric nervous system of the gut as well as the extensive microbial populations located within the GI tract, it is plausible that the structure and function of GALTs may be impacted when neural development goes awry, such as in cases of neurodevelopmental disorder. Indeed, changes to the GALT-microbiota-gut-brain signalling circuitry may be visually undetectable, yet may still lead to dysfunction in gut signalling and nervous system homeostasis.

### Mouse Peyer’s patch development

In mice, Peyer’s patch lymphoid aggregates form along the embryonic small intestine in random locations between 12.5 and 13.5 days post coitum (dpc). Throughout foetal development in mice, chemokines such as CXCL12 and -13, CCL19, -20 and -21, and adhesion molecules including vascular cell adhesion molecule 1 (VCAM-1), mucosal vascular addressing cell adhesion molecule 1 (MAD-CAM1) and intercellular adhesion molecule 1 (ICAM-1), altogether signal to promote the circulation of LTi cells and the migration of T- and B-lymphocytes into primordia resembling Peyer’s patches (Kunkel et al. 2003; Patel et al. 2012). Within the mucosa, lymphocytes are redistributed into distinct functional compartments. For example, B-cells form the follicles of the germinal centres while T-cells form the interfollicular zones. During postnatal development, exposure to foreign antigens is critical to the maturation of Peyer’s patches, in terms of its structure and function, as well as the development of internal germinal centres. Such observations have been derived from studies of germ-free mice where Peyer’s patches are present but remain immature prior to bacterial colonisation, whereby the size and cellular composition of Peyer’s patches changes when the gastrointestinal tract of germ-free mice is colonized by microbes (Chung et al. 2012). Studying the complex interactions of the host and commensal microbiota and how these shape developmental processes, host homeostasis and GALT neuroimmunomodulatory functions will support our improved understanding of such mechanisms in neurodevelopmental disorders such as autism.

### Mouse caecal patch development

Although little is known about the process or timing of caecal patch development in mice, caecal patch morphology has recently been examined in 8-week-old C57Bl/6 wild type mice and littermates expressing the autism-associated R451C missense mutation in the *Nlgn3* gene encoding the Neuroligin-3 neuronal cell adhesion molecule (Sharna et al. 2020). This study showed subtle changes in the morphology of Iba-1 labelled macrophages correlated with the presence of the R451C mutation, suggesting an increased immune response at baseline in mutant mice and effects on the structure (Hosie et al. 2019; Sharna et al. 2020) and function (Hosie et al. 2019) of the postnatal mouse ENS. This finding thus provides motivation for further studies exploring the underlying mechanistic, developmental changes in caecal GALT that influence autism endophenotypes in this preclinical model of autism.

In contrast to the paucity of studies with mice, there is significant literature on rabbit caecal patch follicles and the migration of lymphocytes into these tissues (Hanson and Lanning 2008). Comparable to Peyer’s patch development, lymphocyte migration into caecal patch tissue continues postnatally upon antigen stimulation and microbial colonisation of the gastrointestinal tract in rabbits. The development of the rabbit caecal patch occurs in two phases. In the initial phase, B cells migrate into the nascent caecal patch follicles, followed by subsequent B cell proliferation, mediated by the intestinal microbiota (Hanson and Lanning 2008). The migration of B cells into the rabbit appendix begins approximately 2 days after birth followed by seeding of appendix follicles for 1–2 weeks which is thought to be regulated by the expression of the peripheral lymph node endothelial adhesion molecule, known as addressin, encoded by the *MADCAM1* gene and expressed in high endothelial venules (HEVs) of the appendix (Sinha et al. 2006).

Although similarities may exist between the development of caecal patch and Peyer’s patch, the precise identity and composition of caecal primordial cell types that underlie structural and functional differences between these two GALT tissues remains to be clarified. Similarly, the mechanism of interaction between each of these two types of GALT aggregates and their respective GI environments and microbial populations remains unclear. Given the significant microbial content of the caecum in mice, the caecal patch may serve a prominent immune-surveillance role, so as to detect invading threats and pathogens. The anatomical location of the caecum may also present as an optimal site for influencing gut health in both the oral and distal lengths within the GI tract. Additionally, studies examining the developmental trajectory and functions of the caecal patch in mice will clarify their putative regulatory functions in gut health and the microbiota-gut-brain axis. When considering factors such as the GI regional environment, adjacent microbial populations and host genetics, the response of caecal patch tissue to immune challenge could differ significantly from that of Peyer’s patches, and their distinct responses and neuro-immune interactions could underpin homeostasis as well as in disease states.

## Initiation of immune responses in the gut

Both Peyer’s patches and caecal patch tissue monitor potential threats deriving from the luminal content of the gastrointestinal tract. Both these regions contain highly organised lymphoid tissue that absorb and process antigens from the luminal content to initiate an appropriate immune response. Peyer’s patches generate protective secretory Immunoglobulin A (sIgA) that influences signalling primarily within the small intestine, whereas caecal patches produce IgA-secreting B cells that migrate both to the small intestine and the colon (Masahata et al. 2014). Within both the Peyer’s and caecal patches, the follicle-associated epithelium is densely populated with **i**ntraepithelial lymphocytes such as T reg cells, M cells and HLA-DR (human leukocytes antigen D related) and T and B cells (Spencer et al. 1985). Such concentrations of immune cells explain why the caecal patch is particularly responsive to immune stimulation (Corr et al. 2008; Uchida 1988).

### Microfold cells

Microfold (M) cells are located in the follicular associated epithelium of Peyer’s and caecal patches and transcytose luminal content. M cells can be morphologically distinguished from intestinal enterocytes based on their short irregular microvilli located at the apical surface, in contrast to the uniform and densely packed microvilli of enterocytes.

The intestinal epithelial cells in the follicular associated epithelium originate from stem cells in crypts positioned between a villus and dome of the Peyer’s patches (Heath 1996). Cells of the crypts, subsequently move into the dome region and differentiate into M cells and absorptive enterocytes (Gavrieli et al. 1992). The basolateral membrane of M cells contains large folds or invaginations which create a shorter transport route for passage of internalized pathogens to awaiting lymphocytes and macrophages (Kraehenbuhl and Neutra 1992; Nicoletti 2000).

Although M cells are adept at targeting pathogens, the precise mechanism by which this occurs is currently unknown. Lymphocytes and macrophages internalise antigens transcytosed by M cells, following which they act as antigen-presenting cells and migrate to the sub-epithelial dome (Gebert et al. 1996). The presence of the chemokine receptor CCR6 is important for the migration of antigen-presenting B cells to the sub epithelial dome. This was demonstrated in studies of bone marrow chimeric mice, whereby CCR6-deficient B cells were incapable of localising to that region (Reboldi et al. 2016). Once in the sub**-**epithelial dome, antigen presenting cells interact with B and T lymphocytes, dendritic cells and macrophages to form part of the mononuclear phagocyte system. In this process, antigen presenting cells present the antigen/class II major histocompatibility complex (MHC-II) and cytokines to naïve lymphocytes. Within Peyer’s patches, B cells undergo an immunoglobulin class switch (i.e., from expressing IgM to IgA) due to the influence of factors such as transforming growth factor-β (TGF-β), and IL-10 (Mowat 2003). These “primed” lymphocytes then exit the Peyer’s patch via the lymphatic system and proceed to the mucosal surface.

### Secretory immunoglobulin A

The production and release of secretory IgA is vital as a first line of defence against pathogenic organisms in the gut. The polymeric structure of IgA interacts with large antigens via multiple epitopes and prevents bacteria and viruses attaching to mucosal cells.

Lysosome-expressing dendritic cells are part of the mononuclear phagocyte system and are also found in sub**-**epithelial dome tissue. Dendritic cells have dendritic-like projections that traverse the transcellular pores of M cells and internalise particles such as bacteria and pathogens (Mowat 2003). These dendritic cells also display the highest surface expression of MHC-II, CD40 and CD80 (Jung et al. 2010). Relevant to neuroimmunomodulatory effects, reducing gastrointestinal innervation by vagotomy decreased secretion of IgA in the small intestine in mice (Pérez-López et al. 2019; Arciniega-Martínez et al. 2019). The activity of GALT in generating an immune response via secretory IgA could therefore also be impacted in neurodevelopmental disorders and contribute to aberrant immune responses.

### Migration of IgA-expressing B cells

In humans, GALT CCR9 chemokine receptor activation generates secretory IgA which mediates migration in the small intestine (Pabst et al. 2004). In contrast, CCR10-mediated secretion is important in secretory IgA-expressing cellular migration in both the small and large intestines in mice (Masahata et al. 2014), with CCR10 being highly expressed in the caecal patch. Germ-free mice, however, show a severe decrease in intestinal secretory IgA cells due to the absence of commensal bacteria.

Secretory IgA antibodies in mucosal secretions promote immune homeostasis by restricting exposure of environmental and microbial antigens to the body and forming the composition of the commensal microbiota (Macpherson et al. 2008). Plasma cells in the intestinal lamina propria secrete IgA which plays an important role by binding to pathogens or pathogen-derived antigens to limit the invasion into the body (Strugnell and Wijburg 2010; Latiff and Kerr 2007). Most IgA mucosal plasma cells are generated from activated B cells in GALT. This process is dependent on the presence of gut microbiota as demonstrated by experiments in germ-free mice that lack significant IgA responses (Brandtzaeg et al. 2008; Macpherson et al. 2015, 2000; Bos et al. 1989). Interestingly, dendritic cells in the caecal patch, but not in Peyer’s patches induce expression of the CCR10 chemokine to mediate the recruitment of plasmablasts to both the small and large intestines (Masahata et al. 2014). The precise roles for caecal patch in gut immunological responses remain to be better characterised.

## Innervation of GALT

GALT plays a central role in maintaining gastrointestinal homeostasis including baseline levels of inflammation, the prevention of microbial overgrowth and neuroimmune interactions. The ENS comprises a complex network of 200–600 million neurons (Furness 2012) and glial cells involved in complex cross‐talk with bacterial, immune and epithelial cells. The intestinal mucosa is densely populated with neuronal fibres in close contact with epithelial cells and immune cells. Studies of human Peyer’s patches reveal peptidergic innervation including Substance P, Vasoactive Intestinal Peptide (VIP) and Calcitonin gene-related peptide (CGRP) immunoreactivity in cells within the dome area (Vulchanova et al. 2007). In 2–5 month-old lambs, labelling for Tyrosine Hydroxylase (TH), dopamine beta hydroxylase (DBH), Choline acetyl transferase (ChAT), Calbindin, Nitric Oxide Synthase and CGRP was identified in close proximity to and inside Peyer’s patch follicles (Chiocchetti et al. 2008). In the mouse, Peyer’s patches are innervated, express VIP (Ottaway et al. 1987) as well as the muscarinic type 2 (M2) cholinergic receptor (Ma et al. 2007) in the dome region of Peyer’s patch tissues. Both Ma and Al-Shalan and coworkers found that innervation was more prominent in the interfollicular region of the Peyer’s patch in mice compared to the follicular region (Al-Shalan et al. 2020).

Interestingly, at baseline conditions, Peyer’s patches are sparsely innervated but express neuropeptide receptors in the presence of inflammation (Stead 1992). The enteric nervous system is thought to play an important role in enabling prion infection which can contribute to several fatal and transmissible neurodegenerative diseases (Chiocchetti et al. 2008). Indeed, Margolis and others describe denser innervation surrounding Peyer’s patches compared with other regions of mucosa in the mouse, and such neuroanatomical features could be indicative of roles for surveillance and mounting an immune response to pathogen infiltration (Margolis et al. 2016a, b). Indeed, the role of the nervous system in protecting from *Salmonella* infection has recently been demonstrated. Nociceptor neurons located in dorsal root ganglia that innervate the ileum regulate the functioning of Peyer’s patches by decreasing the density of M cells (Lai et al. 2020). Lai et al., showed that detection of *Salmonella* by these neurons promotes their release of CGRP peptide within Peyer’s patches. This regulation of M cells maintains the colonisation of segmented filamentous bacteria in the lumen, providing a defence from *Salmonella* infection.

## The role of microbes in Peyer’s and caecal patch function

Peyer’s patch function is regulated by the constant interaction of food and microbial-derived antigens with germinal centres. Peyer’s patches are present in germ-free mice, but they are reduced in size. “Humanised mice” as recipients of human microbial colonisation have germinal centres of Peyer’s patches that are reduced in size compared to mice receiving mouse microbial transplants (Chung et al. 2012). These findings indicate that the host species of which the microbial community is derived is important for shaping GALT (Chung et al., 2012). In addition, germ-free mice showed increased weight and size of the caecum in comparison to specific pathogen free and colonised mice (Smith et al. 2007). A study comparing germ-free mice and specific pathogen-free mice also showed a reduction in the size of the caecal patch region in germ-free mice, indicating that, similar to Peyer’s patch tissue, the development of the caecal patch is influenced by commensal microbiota (Masahata et al. 2014). Studies exploring the microbiome of the caecum have identified defined the structure and function of the caecal microbiome which undergoes a major disturbance in response to starvation, antibiotics and hepatectomy (Zaborin et al. 2020).

Biofilms are integrated communities of microbes immersed within surface-associated matrices. Based on cell surface marker expression of microbiota, biofilms are part of typical gut microbiota (Banwell et al. 1985; Banwell et al. 1988; Macfarlane et al. 1997; Cassels and Wolf 1995). Within the gastrointestinal tract, these structures enable resistance to hydrodynamic shear forces (Liu and Tay 2002). In addition, symbionts inhabiting the polysaccharide-rich mucus gel layer overlaying the gut epithelium build a biofilm-like community of microbes that benefit the host by boosting immune responses and digestion of luminal contents (Sonnenburg et al. 2004). Given that multiple studies in human subjects have shown microbial dysbiosis in neurodevelopmental disorders (reviewed in (Alamoudi et al. 2022)), changes in GALT function and the mucus biofilm environment may be relevant to disruptions in homeostatic mechanisms and the immune response in gut observed in autism.

## Gastrointestinal histopathology in preclinical models

Gastrointestinal dysfunction is a common co-morbidity observed in a range of neurodevelopmental disorders including ASD. GI symptoms commonly experienced by individuals diagnosed with ASD include constipation, diarrhoea, abdominal pain, and more (Holingue et al. 2018), however structural and functional changes in the GI tract are less well documented. In preclinical models of autism, GI changes including disturbed morphological structures in the small intestine and colon, as well as altered mucosal permeability have been identified (Margolis et al. 2016a, b; Hosie et al. 2019; Sauer et al. 2019; Leembruggen et al. 2020). Margolis and colleagues investigated GI function in mice expressing an autism-associated missense mutation resulting in the substitution of an alanine residue for glycine at position 56 of the serotonin transporter (SERT G56A). They found that SERT G56A mice had reduced small intestinal villus height as well as shorter small intestinal and colonic crypt depths plus increased mucosal permeability (Margolis et al. 2016a, b). Similarly, Sauer and co-workers found that Shank3 knock-out mice had shorter small intestinal villi, however, crypt depth remained unchanged (Sauer et al. 2019). Interestingly, Neuroligin-3 knock-out mice showed no differences in villus height or crypt depth in the jejunum and colon (Leembruggen et al. 2020). In contrast, although histological changes in Neuroligin-3 knock-in mice expressing an arginine to cysteine modifying missense mutation at position 451 on the protein (NL3^R451C^ mice) have not been reported, microbial dysbiosis in faecal samples were identified (Hosie et al. 2019). Furthermore, a previous study in NL3^R451C^ mice indicated an increase in the density of Iba-1 labelled macrophages within the GALT (caecal patch) and that these cells had a more rounded morphology, potentially indicating an activated cellular phenotype (Sharna et al. 2020). Currently, it is unknown whether similar changes in GALT are present in other mouse models of ASD. Investigations of morphological and functional changes to the epithelium lining of the gastrointestinal tract as well as shifts in microbial populations could point to disrupted structure and function of GALT in other ASD preclinical models. As discussed throughout this review, GALT plays an important role in maintaining barrier integrity by surveying luminal contents and interacting with immune cells to maintain physiological homeostasis of the host. GALT dysfunction can also affect mucosal permeability, a commonly observed alteration in ASD rodent models (Chandran et al. 2003; Barreau et al. 2007; Chatterjee et al. 2022). Further investigations are necessary to explore GALT morphology and function in ASD animal models to better understand their role in GI dysfunction in ASD patients.

## Conclusion

GALT is an important component in sensing and responding to the microbial environment and therefore should be considered as a major component contributing to microbiota-gut-brain axis function. Alterations in the nervous system occurring in neurodevelopmental disorders may also impact GALT activity as well as gastrointestinal mucus production and composition, epithelial replenishment and the distribution of microbial populations along the gastrointestinal tract (Herath et al. 2020). These factors could potentially contribute to gut symptoms and modification of microbial populations, changes that have been observed in the context of autism spectrum disorder. Neuroimmunodulatory interactions involving GALT need to be further investigated to unravel the contribution of microbiota-gut-brain axis pathways in neurodevelopmental disorders including autism.
